# Prevalence and radiographic characteristics of supernumerary teeth in a Saudi population: A retrospective CBCT-based study

**DOI:** 10.12669/pjms.42.1.12457

**Published:** 2026-01

**Authors:** Shaul Hameed Kolarkodi, Ebrahim Alshawy

**Affiliations:** 1 Shaul Hameed Kolarkodi Department of Oral and Maxillofacial Diagnostic Sciences, College of Dentistry, Qassim University, Qassim, Saudi Arabia; 2 Ebrahim Alshawy Department of Orthodontics and Pediatric Dentistry, College of Dentistry, Qassim University, Qassim, Saudi Arabia

**Keywords:** CBCT, Conical teeth, Dental anomalies, Diagnostic imaging, Hyperdontia, Impaction, Inverted orientation, Prevalence, Saudi population, Supernumerary teeth

## Abstract

**Objectives:**

This study aimed to determine the prevalence and radiographic characteristics of supernumerary teeth (ST) in a Saudi population using cone beam computed tomography (CBCT). The research also explored the clinical and orthodontic implications of ST on tooth eruption and treatment planning.

**Methodology:**

A retrospective cross-sectional study was conducted at the Department of Diagnostic Sciences, College of Dentistry, Qassim University. A total of 992 CBCT scans obtained between January 2020 to January 2024 were reviewed. Demographic and radiographic variables-such as morphology, orientation, eruption status and relationship with adjacent teeth-were recorded in Microsoft Excel. Two calibrated oral radiologists independently evaluated the scans. Statistical analysis, including descriptive statistics and chi-square tests, was performed using SPSS version 27.0.

**Results:**

Supernumerary teeth were detected in 28 patients, indicating an overall prevalence of 2.82%. The prevalence among males (5.61%) was significantly higher than in females (1.10%). Most individuals presented with a single ST (67.86%). The common morphologies were conical (46.15%) and supplemental (38.46%). A majority were impacted (76.92%) and exhibited an inverted orientation (33.33%). In 20.51% of cases, ST obstructed the eruption of adjacent permanent teeth.

**Conclusions:**

Supernumerary teeth in this population showed a strong male predilection and were predominantly conical, impacted and inverted. CBCT was instrumental in their detailed assessment.

## INTRODUCTION

Supernumerary teeth (ST), also known as hyperdontia, refer to the “Presence of teeth in addition to normal dentition.” These teeth can exist in primary and permanent dentitions and can be in the form of isolated or clusters. Whereas these anomalies are often asymptomatic, they are connected to a range of clinical complications such as delayed eruption, malocclusion, root resorption of adjacent teeth, cystic lesions, and crowding, which is why their detection and early intervention is important to achieve good patient outcomes.^1^,^2^ Although their exact etiology is still under full understanding, a multifactorial interaction between inheritable elements and environmental factors is believed to contribute to the onset of such anomalies.^3^ Their prevalence and morphological patterns vary significantly across populations and age groups. Males are more affected than females and the maxillary area especially the anterior part is the most affected site and causes supernumerary teeth which interfere with the eruption of permanent dentition and results in diastemas, midline deviations, crowding, and impaction and thus make the treatment procedures complicated and the treatment longer.^4^,^5^ Timely identification is thus necessary in order to reduce the complexity and duration of treatment.^6^

The conical shape is the most commonly seen morphological variant, and the teeth will be frequently unerupted or impacted. This leads to the need of an advanced imaging modality to detect and characterise accurately. The use of cone-beam computed tomography (CBCT) has become the new method of imaging used to assess supernumerary teeth, due to their high ability to give three dimensionality of the position of teeth, the orientation, the morphology of teeth and the proximity of the tooth to the neighboring anatomical structures. CBCT can be used in the planning of surgical and orthodontic procedures as well as in the diagnosis of the conditions.^7^

Substantial studies using CBCT have addressed the frequency and the nature of supernumerary teeth in different populations. A large-scale study in China by Jiang et al. (including more than 60,000 individuals) reported a prevalence of 1.5% supernumerary teeth, the majority of which were conical, impacted, and located in the anterior of the maxilla.^5^ Similarly, a study by He et al. in a pediatric Chinese cohort highlighted the eruption potential of ST and demonstrated the influence of age, orientation and anatomical position on eruption outcomes.^8^ Mossaz et al. reported in their CBCT study that the diagnostic value of 3D imaging in the detection of complications of supernumerary teeth, especially in supernumerary premolars.^4^ Despite these, there are only a few studies investigating supernumerary teeth in the Saudi population, especially with the use of CBCT. The need to have regional genetic diversity and benefits provided by three-dimensional imaging presents a need to have localized data that can be used to inform clinical practice. The current research thus aimed to assess the prevalence, distribution, and radiographic appearance of supernumerary teeth in a Saudi cohort of studies based on the use of CBCT.

## METHODOLOGY

This research was a cross-sectional retrospective study undertaken at the Department of Diagnostic Sciences, College of Dentistry, Qassim University, Saudi Arabia. The main aim of the study was to establish the prevalence and radiographic features of supernumerary teeth with CBCT.

### Ethical approval:

It was obtained from the Institutional Review Board of Qassim University (Order No. 23-41-06; Dated: June 5, 2023), with all procedures conducted in accordance with institutional guidelines and the Declaration of Helsinki

. The sample included CBCT scans taken between January 2020 and December 2024 on patients visiting the diagnostic clinics of the College of Dentistry, Qassim University with different dental indicators, such as orthodontic assessment, implant planning, impacted tooth evaluation, and pathology investigation. A total of 992 CBCT scans were analyzed, which were divided into five years, 130 in 2020, 190 in 2021, 202 in 2022, 260 in 2023, and 210 in 2024. Since the research used a referral based clinical population, the slight changes in annual prevalence might simply be caused by differences in patient inflow, referral trends and departmental workload and not necessarily caused by actual changes in the population.

### Inclusion & Exclusion criteria:

Saudi citizens aged six years and above who had high-quality and complete arch CBCT images that gave full visualization of both jaws. Scans were excluded when they had motion artifact or incomplete coverage of the maxillofacial region, when the patients had a history of trauma or surgical intervention or extractions in interest, when they had syndromic or general systemic associations known to be accompanied with multiple supernumerary teeth, including cleidocranial dysplasia, Gardner syndrome, Down syndrome or cleft lip and palate.^9^ Syndromic cases were excluded by looking into the clinical records of each patient and his/her radiographic features. The cases with generalized skeletal anomalies, multiple unerupted or malformed teeth or any record of genetic disorders in medical history were excluded so that only non-syndromic individuals were used in the analysis.

All CBCT scans were made with a standardized imaging protocol, having a voxel size of 0.16mm, a field of view of 15 ˣ 8.5 cm, slice size of 1mm. Scans were obtained using a Sirona Dental Systems unit (Sirona Dental Systems GmbH, Bensheim, Hessen, Germany) operated with Sidexis-XG software under exposure parameters of 98 kVp, 3–6 mA, and a clinical exposure duration of 2–5 seconds, followed by reconstruction of approximately 14seconds. Any DICOM data was anonymized and viewed on specific CBCT viewing software which enabled multiplanar reconstruction and three-dimensional representation. All the volumes were opened in an axial, coronal and sagittal position and head placement was standardized with the use of the Frankfort and mid-sagittal position to ensure consistency. In the case of potential supernumerary teeth (ST) or abnormalities, a general scan of the entire slices was initially done and then a systematic region-wise analysis was done on the maxillary and mandibular arch in the anterior, premolar and molar region sequence. Slices were evaluated at 1 mm thickness with fine scrolling (≤0.5–1 mm) to ensure no structures were missed.

Suspected supernumerary teeth were confirmed by cross-checking continuity in all three planes to differentiate true teeth from artifacts or calcifications. Three-dimensional rendered and maximum-intensity-projection (MIP) views were used where necessary to clarify the morphology, position, and orientation of the teeth relative to adjacent structures. For each confirmed ST, detailed information was recorded on a standardized data sheet, including anatomical location (maxilla or mandible; anterior, premolar, or molar region), morphology (conical, tuberculate, supplemental, or odontoma-like), orientation (vertical, inverted, horizontal, or oblique), spatial position (buccal, palatal/lingual, or central), eruption status (erupted or impacted), and relationship with adjacent teeth (no relation or obstructing eruption).

Orientation definitions were applied consistently, vertical teeth had their long axis parallel to neighboring teeth, inverted teeth had crowns directed opposite to the normal orientation, horizontal teeth were nearly perpendicular to the normal tooth axis, and oblique teeth lay between vertical and horizontal alignment. Additional findings such as root resorption, cystic change, or displacement were noted qualitatively but not analyzed statistically because of incomplete clinical confirmation.

The calibrated measuring instruments in the viewing software were used to acquire linear and angular measures (isotropic voxel=0.16mm). The two study authors (SHK and EA) reviewed all the scans independently, whereby, SHK and EA were an oral and maxillofacial radiologist and orthodontist, respectively. The joint consensus review was utilized to solve disagreements. To determine quality assurance, 50 randomly chosen scans were re-examined after two weeks to determine intra- and inter-examiner reliability by means of Cohen 6 coefficient with 95% confidence intervals.

Besides the measurement of morphology, orientation and eruption state, every CBCT scan was also investigated in terms of potential pathological correlation, such as root resorption of neighboring teeth, cystic alterations or displacement. Nevertheless, since the clinical records and the histopathological confirmations were not complete in various cases, they were only described descriptively and were not factored in the quantitative analysis.

Radiographic evaluations were independently performed by the two study authors (SHK and EA). Before commencing data collection, both examiners participated in a structured calibration session to standardize the diagnostic criteria and classification protocol for identifying supernumerary teeth on CBCT images. To assess intra-examiner reliability, each author re-evaluated a random subset of 50 CBCT scans after a two-week interval under identical viewing conditions. Inter-examiner reliability was determined by comparing both authors’ initial evaluations of the same set of scans. The 95% confidence interval (CI) of the Cohen 3 coefficient of agreement was used to determine the level of consistency between observations in the detection and classification of supernumerary teeth. The k values were explained based on the standards of Landis and Koch (1977): 0 = low, poor; 0.01-0.19 = slight; 0.20-0.40 = fair; 0.40-0.60= moderate; 0.60-0.80 = substantial and 0.80 -1= almost perfect agreement.

### Statistical analysis:

All the demographic and radiographic data were documented in an organized data collection sheet. Findings were summarized using descriptive statistics such as frequencies, percentages, means and standard deviations. The analysis of the data was carried out with the help of standard statistical software (SPSS version 27.0).

## RESULTS

To ensure reliability and reproducibility of the radiographic evaluations, inter- and intra-examiner agreement were assessed using Cohen’s kappa (k) statistics. The inter-examiner k value for the detection and classification of supernumerary teeth was 0.87 (95% CI: 0.79-0.95), indicating almost perfect agreement. The intra-examiner k values for SHK and EA were 0.91 (95% CI: 0.84-0.98) and 0.89 (95% CI: 0.82-0.96), respectively, confirming a high level of consistency in evaluations between and within examiners.

A total of 992 CBCT scans were evaluated from the years 2020 to 2024 to assess the presence and distribution of supernumerary teeth. The prevalence rate among the male participants was 5.61% which is considerably higher than the prevalence rate of the females who was 1.10%. The frequency and annual changes of the prevalence of single and multiple supernumerary teeth are recorded.

The distribution of the cases in an annual basis with the number of affected persons, the form of supernumerary teeth (single or multiple) and the prevalence rate per year is shown in Table-I. The 992 scans were analyzed to show that there was an average prevalence of 2.82 in the population studied of supernumerary teeth. Of the 28 cases found, 19 patients (1.92^−^ 1) had one supernumerary tooth, and 9 patients (0.91 -1) had more than one supernumerary tooth. The peak prevalence was in 2020 (3.85%), and then it decreased slightly in the following years. The information continuously showed that single supernumerary cases had a greater proportion than multiple cases in all years.

**Table-I:** Yearly Distribution and Prevalence of Supernumerary Teeth Cases.

Year	Total number of cases	Patients with single supernumerary teeth	Patients with Multiple supernumerary teeth	Total number of participants examined	Prevalence of cases in each year Total	Prevalence of patients having Single SNT	Prevalence of patients having Multiple SNT
2020	5	4	1	130	3.85	3.08	0.77
2021	6	4	2	190	3.16	2.11	1.05
2022	5	4	1	202	2.48	1.98	0.50
2023	7	4	3	260	2.69	1.53	1.15
2024	5	3	2	210	2.38	1.42	0.95
Grand Total	28	19	9	992	2.82	1.92	0.91

The most common (46.15%) were conical-shaped supernumerary teeth, then came the supplemental types (38.46%), and lastly round and tuberculate shaped. Table-II. Most of the supernumerary teeth were affected (76.92%), which means that they did not erupt into the mouth. The percentage of erupted teeth is only 15.25, as per which the majority of the supernumerary teeth are still in the bone and might need radiographic diagnosis. About 79.48 -1 of the supernumerary teeth were not correlated with adjacent teeth, and the remainder 20.51 -1 were observed to interfere with the eruption of permanent teeth, and a possible clinical complication may or may not require action. The most common orientation was inverted (33.33%), then vertical positioning (23.07%), and then palatal positioning (20.54%). Other orientations that were less popular were horizontal (10.25 0.769), oblique (7.69) and rare orientations (labial, between adjacent teeth 2.56 2.56).

**Table-II:** Distribution of Morphological, Positional and Eruption Characteristics of Supernumerary Teeth.

Characteristic	Category	Frequency (n)	Percent (%)
Shape	Conical	18	46.15
	Round	2	5.12
	Tuberculate	4	10.25
	Supplemental	15	38.46
Tooth Status	Impacted	30	76.92
	Erupted	9	23.08
Relation to Eruption	No relation	31	79.49
	Obstructing the eruption of permanent teeth	8	20.51
Alignment & Position	Vertical	9	23.07
	Inverted	13	33.33
	Horizontal	4	10.25
	Oblique	3	7.69
	Labial	1	2.56
	Palatal	8	20.54
	In Between	1	2.56

The presence of the supernumerary teeth was predominantly distributed on the maxillary arch with 61.5 % of all cases being based on the anterior region. Table-III.This observation supports the existing agreement that the premaxillary region, particularly the incisor region, is the most common area of supernumerary development. The maxillary premolar and molar/distomolar areas were 17.9% and 5.1%, respectively. The mandibular arch showed much lesser involvement (15.4% overall) and mostly was within the premolar area (10.3) and rarely molar area (5.1). In the anterior cases, mesiodens were reported to account for almost 43.5, thus demonstrating that they were the most common type of supernumerary teeth.

**Table-III:** Distribution of Supernumerary Teeth by Jaw and Region

Jaw/Region	Frequency (n)	Percentage (%)
Maxillary anterior	24	61.5
Maxillary premolar	7	17.9
Maxillary molar/Distomolar	2	5.1
Mandibular premolar	4	10.3
Mandibular molar	2	5.1
Total	39	100

Regarding spatial orientation, most of the supernumerary teeth were inverted (38.0 %) then vertical (28.2%), horizontal (17.9%) and oblique (7.7%). These trends reveal that most supernumerary teeth deviate from the normal eruption axis and usually cause impaction and eruption disruptions. Their hidden nature (preponderance of palatal positioning 20.53%) is further evidenced by palatal positioning and often requires higher imaging like CBCT to accurately locate them.

**Fig.1:**
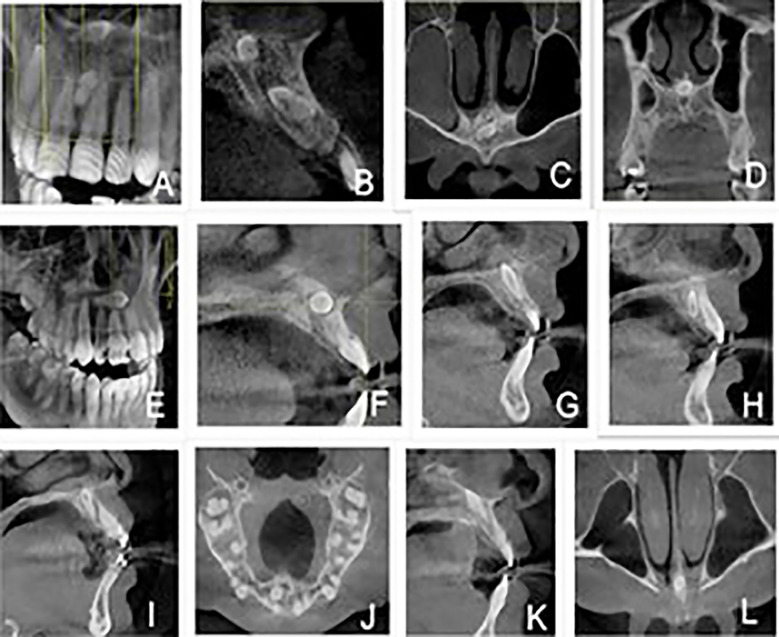
Cone Beam CT Images Depicting Variations in Mesiodens Presentation Including Number, Orientation, Location and Anatomical Relations

**Fig.2:**
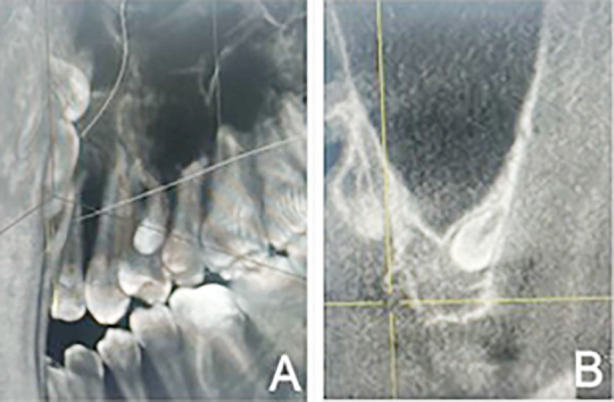
A & B showing round-shaped supernumerary tooth.

**Fig.3:**
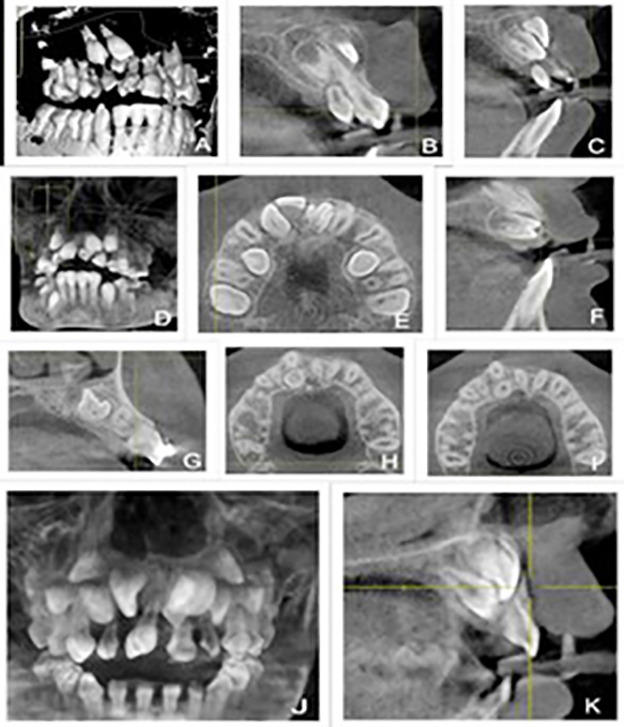
CBCT Representations of Tuberculate Supernumerary Teeth Showing Pulp Horn Variations and Root Development Stages.

**Fig.4:**
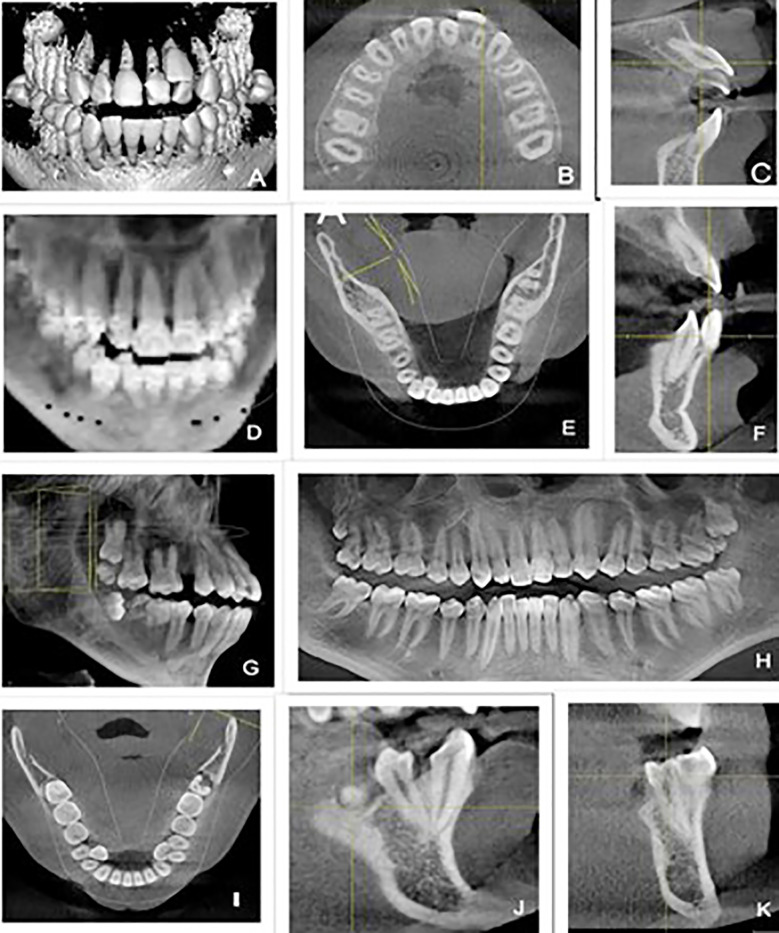
CBCT and Panoramic Views Demonstrating a Range of Supernumerary Teeth with Variations in Number, Location, Morphology, Orientation and Developmental Stages.

Fig.1 reveals individual mesiodens cases illustrating differences in quantity, positions (vertical, inverted, horizontal, and oblique), and location in the anatomy (nasopalatine and palatal), which are in relation to their surrounding structures like nasopalatine canal. Fig.2A and Fig.2B show specifically round-shaped supernumerary teeth which were a minor proportion of morphologies in the sample. Fig.3 shows examples of tuberculate supernumerary teeth, where there are variations in the number of pulp horns (two or three or more) and stages of root development including the incomplete apexogenesis, and a rare instance of complete root development. Fig.4 gives us a systematic visual summary of supernumerary teeth and the variability in the number, morphology, anatomical positioning and developmental stages in various patients (Fig.1 to Fig. IV).

## DISCUSSION

In the current retrospective cross-sectional, CBCT-based research, the authors investigated the prevalence and nature of supernumerary teeth (ST) in a Saudi population at Qassim University. Out of 992 participants who had been assessed in 2020-2024, 28 were found to have supernumerary teeth, which represents a prevalence of 2.82%. This has been shown to be slightly higher than the 2% prevalence observed by Mallineni et al. and the 1.8% prevalence observed by AlHumaid et al. both studies that were in cohorts in Saudi Arabia, respectively, of 11 and 12 years of age.^10^,^11^ This prevalence is less than 3.9% prevalence of orthodontics in a Pakistani orthodontic population but significantly higher than the prevalence of 0.2% prevalence of distomolar reported by Niazi et al. and 1.2% prevalence of distomolar reported by Gichki et al. in the Pakistani samples.^12^–^14^ The variability between the international populations is quite well-documented, with Thailand reporting 1.2% of variation, white Europeans reporting 3% of variation, and Chinese population reporting significantly higher rates, 10.52% and 10.87% respectively.^15^–^18^ Considered in the context of the larger literature in oral anomalies in Pakistan like Masood et al. the present study findings have specific information regarding hyperdontia, similar to international surveys by Kashmoola et al. 19,20 The reported adjusted prevalence in the review of panoramics radiograph studies by Anthonappa et al., who reported prevalence rates between 2.4% . and 6%. after accounting for imaging sensitivity limitations, agree with the current findings.^21^

The main observation of the current research is the extreme male predominance. 35 out of 624 males (5.61%), and 4 out of 363 females (1.10%), had supernumerary teeth, meaning that it was much higher in males. This gender difference is supported by recent extensive research, such as that of the Saudi study by Mallineni et al. (76% male), Henninger et al. (1.65:1), Ma et al. (1.86:1) and Xue et al. (2.30:1 ratio).^10^,^16^,^17^,^22^ The present findings are in accord with the basis literature provided by Ata-Ali et al., Mossaz et al., and Mahabob et al., who also indicated higher frequency of ST in males.^1^,^4^,^23^ A contrasting Pakistani study by Amin had a ratio of 2:1 females to males, and Hou et al. had a female bias of slightly higher than a 6:5 ratio in mandibular ST, indicating that the anatomic position could be a determinant of gender distribution, at least in mandibular ST.^24^ Though in studies like Demiriz et al. female occurrence has been observed, the general trend in the literature is in favour of male predisposition, which can be attributed to a genetic or hormonal effect on the development of teeth in males.^25^

The conical type (46.15%) of the supernumerary teeth was the most widespread and was accompanied by the supplemental type (38.46%). Recent literature is very much supportive of this distribution, the current study findings are similar to Mallineni et al. which acquired a result of 60% conical morphology and large CBCT cohorts by Xue et al. which also found conical as the most common type. 10,22 This is consistent with earlier literature, such as Jiang et al., Gürler et al., Syriac et al., and Khandelwal et al., who all reported conical morphology as the predominant form.^5^,^7^,^26^,^27^ The shape of distomolars which Gichki et al. found in a Pakistani sample was mainly tuberculated. 14 On the other hand, Anny et al. described supplemental-type teeth to be most prevalent (76.7%) in a Thai population. 15

Concerning eruption status, most of the supernumerary teeth in the present study were affected (76.92%). This observation is similar to the rest of the recent discoveries, where high impaction rates are also noted by Henninger et al. (74%), Mallineni et al. (66%), Anny et al. (70%), and Hou et al. (96.77% in the mandibles).^10^,^15^,^16^,^24^ Similar trends were also reported in earlier studies by Jiang et al. and Delilbasi et al.^5^,^7^ On orientation, most frequently used (33.33%) was inverted orientation, which is a crucial diagnostic feature which is backed by extensive CBCT studies by Xue et al and Jiang et al.^5^,^22^ Contrarily, Mallineni et al. and Henninger et al. found a range of positional variability with normal orientation as predominant in their groups, indicating high variability of the positioning outcome. 10,16 Previous research works by Mossaz et al. and Syriac et al. affirmed that affected ST often possess vertical or inverted orientations, which is the focus of orthodontic importance.^4^,^26^

Anatomically speaking, the current investigation found a strong preference toward the maxillary anterior area where it represents 61.55% of the cases. This is in line with the existing literature, with Xue et al. (89.30% prevalence in central maxillary incisor sites) having the highest instances, but also the results of Ma et al. and Mallineni et al.^17^,^22^,^28^ In comparison, the present paper has reported supernumerary teeth in the mandibular premolar (10.3) and molar (5.1) areas- posterior positions, but less common, that have unique diagnostic issues. Hou et al. described 22 mandibular cases but most of them occur on lingual surface and are near the mental nerve canal, as opposed to the findings made by Gurniak et al. and Gichki et al. that support the molar associations.^14^,^24^,^29^ This contrasts Anny et al. indicated a better prevalence (50%) of the mandibular parapremolar region in a Thai cohort.^15^

One such critical clinical aspect that can be brought out through the data is the relationship between the supernumerary teeth and adjacent dentition. The partial or total blockage of permanent tooth eruption was found in about 20.51% of the studied cases, which is of great clinical significance. This phenomenon was specially covered by a systematic review of Seehra et al. (2023), who concluded that extraction of supernumerary teeth with orthodontic traction provided better incisor eruption success (96.9%), compared to extraction only (57.6%).^30^ They also found that the conical morphology gave greater chances of eruption.^30^ Although our cohort reported an obstruction, root resorption is also reported as an important complication in the literature. 4,31 A CBCT study by Li et al. further took a step forward to risk-prediction of ST-related root resorption, which involves the predictor variables of patient age and root development.^32^ In the isolated case studies, rare sequelae, including fusion between a distal molar and a third molar, have been reported, which complicates the extraction processes.^33^ While our study did not specifically assess cyst formation or root resorption, the high rate of impaction and malposition indicates the importance of early detection and monitoring.

The inclusion of the supernumerary teeth has a significant clinical implication as per an orthodontic point of view. Vahid-Dastjerdi et al. documented more prevalence rates of ST in patients with Class-III malocclusion and a low number in Class-II cases.^34^ This correlation indicates that supernumerary dentition could be the reason behind skeletal and dental development, which requires more complex orthodontics in specific malocclusion phenotypes. Affected supernumerary teeth may also hinder the movement of orthodontic teeth, increase the treatment time, and require surgery on the affected teeth to attain the most optimal results. This problem is especially relevant because orthodontic procedures are becoming more sophisticated and require advanced tools, including micro-implant anchorage, to work with in complicated cases.^35^

The occurrence of supernumerary teeth may coincide with other developmental anomalies. The current analysis, together with a Pakistani cohort of Amin et al., examined concomitant hypo-hyperdontia (CHH), and its link to the loss of third molars, which was preceded by Varela et al.^12^,^36^ Although the association was statistically irrelevant in the Amin cohort, co-occurrence of anomalies is well corroborated. Zengin et al. found out a strong association between the presence of supernumerary teeth and enamel pearls.^12^,^18^ Other Pakistani publications have provided explanations of the common occurrence of the talon cusp and taurodontism hence contributing to the overall information on localized dental anomalies.^37^,^38^ Shoaib and Ahmed also illuminate the management strategies of tooth agenesis, which is an opposite of hyperdontia.^39^

In case of solitary versus multiple supernumerary teeth distribution, 67.86% of the patients in the group reported a single supernumerary tooth whereas 32.14% reported multiple occurrences. This distribution corresponds well to the results revealed by Mallineni et. al. (mostly single, 24.19% two ST), Xue et. al. (64.19% single) and Henninger et. al. (64.19% single).^10^,^16^,^22^ The same trends are observed in other population research, such as those by He et al. and Jiang et al.^5^,^8^ Several supernumerary teeth are relatively rare and often syndromic as demonstrated by numerous cases of unerupted supernumerary in cleidocranial dysplasia reported by Ahmed.^9^ The present study examined a non-syndromic cohort, but other non-syndromic reports of multiple supernumerary teeth at the premolar area are rewarded, including the case reported by King et al.^40^

The current study provides CBCT-based objective data on the occurrence and radiographic characteristics of the supernumerary teeth in a cohort study in Saudi, which fills a significant gap in the literature of the area. Although CBCT has the ability to provide a detailed visualisation of the surrounding anatomical structures, the study could not perform statistical analysis of the related pathologies like root resorption or cystic lesions. Though some of the scans showed radiographic appearances that could have suggested these changes, the nature of the dataset, which was retrospective and no clinical records were available to support them made it impossible to have been certain. The sample was also obtained in one referential institution, and this will limit the ability of the study to generalise the results to the rest of the population. Further, the research specifically used non-syndromic cases, to focus on the non-syndromic population, it is imperative as the prevalence and management among syndromic patients, such as the cleft lip and palate patients are recorded to show high prevalence of dental deviation.^41^ Although CBCT is more accurate in diagnosing as compared to the conventional radiographs, the initial scans do not serve diagnostic but rather research purposes and no additional exposure of the scans was granted. The unquestionable benefit of CBCT in three-dimensional localisation is one of the main strengths, which Mallineni et al. and the classification study of Yifang et al. confirm.^28^,^42^ CBCT is essential in the proper diagnosis and surgical planning as demonstrated in clinical case series by Al-Sehaibany and Nematolahi et al.^43^,^44^ Despite the fact that CBCT provides the best morphological data, the future studies need to include histological analyses to compare the micro-structural constitution of supernumerary and orthodromically developed teeth as it was described by Stefania et al. and this approach might provide some new findings in the field of etiopathogenesis and functional outcome.^45^ All radiographic analyses were also followed according to the principle of ALARA (As Low As Reasonably Achievable), which kept the radiation levels within the clinically justified situations. It is suggested that prospective, multicenter studies using larger and randomly chosen samples and combined clinical-histopathological data are needed to support and expand the present results.

## CONCLUSION

This CBCT based study established the prevalence of 2.82 per cent of supernumerary teeth in a Saudi cohort with a male preponderance and typical characteristics such as conical morphology, inverted placement, and maxillary impaction. Cone-beam computed tomography was better than the two dimensional imaging used in visualizing supernumerary tooth morphology and spatial orientation. Clinically, ST may disrupt eruption, cause malocclusion and extend orthodontic treatment. Early CBCT diagnosis enables early intervention, reduces complications, and educates the treatment planning and planning especially when there is unexplainable crowding or delayed eruption of a tooth.

### Recommendations:

In future are multicenter studies with increased sample size and follow-ups should be conducted to increase generalizability. Future studies should also be done on the development of more developed diagnostic modalities, including convolutional neural network-based radiographic identification of artificial intelligence. Prolonged data would help to determine the enduring effect of supernumerary teeth, whereas the combination of radiological, clinical, and genetic data could help sharpen etiological knowledge and aid in the development of the individual approach to treatment.

### Authors Contribution:

**SHK and EA:** Conceived, designed and conducted the data collection, statistical analysis, manuscript writing, editing and review; approved the final version of the manuscript; and were responsible and accountable for the accuracy and integrity of the work.
